# Corneal nerve loss in adolescents with obesity and acanthosis nigricans

**DOI:** 10.1371/journal.pone.0309761

**Published:** 2024-10-21

**Authors:** Hoda Gad, Hajar Dauleh, Shiga Chirayath, Rasha Amin, Maheen Pasha, Einas Elgassim, Basma Haris, Ghassan Mohamadsalih, Sari Jolkka, Roshirl Biglang-awa, Erlinda Cuatrona, Gina Inso, Gerald Razon, Mohamed A. Hendaus, Farah Wahbeh, Fatima Sajjadi, Yasmeen Al-Hashimi, Noor AlNassr, Ioannis N. Petropoulos, Georgios Ponirakis, Khalid Hussain, Rayaz A. Malik

**Affiliations:** 1 Research Department, Weill Cornell Medicine-Qatar, Doha, Qatar; 2 Endocrinology Department, Sidra Medicine, Doha, Qatar; 3 General Pediatrics Department, Sidra Medicine, Doha, Qatar; 4 Institute of Cardiovascular Medicine, University of Manchester, Manchester, United Kingdom; Muhimbili University of Health and Allied Sciences School of Medicine, UNITED REPUBLIC OF TANZANIA

## Abstract

**Background/Aim:**

Obesity and related metabolic abnormalities in adults are associated with peripheral neuropathy. Acanthosis nigricans (AN) is associated with insulin resistance, fatty liver, hyperlipidemia and glucose intolerance, all of which are risk factors for neuropathy. The aim of this study was to investigate if obese adolescents with AN have evidence of small nerve fiber damage.

**Material and methods:**

Adolescents with obesity with and without AN underwent body composition analysis, assessment of vibration perception threshold (VPT), monofilament sensitivity and corneal confocal microscopy (CCM) to quantify corneal nerve fiber density (CNFD), branch density (CNBD), length (CNFL) and inferior whorl length (IWL).

**Results:**

Forty-six participants with obesity with (n = 31) and without (n = 15) AN aged 15(14–17) years were compared to 20 healthy controls aged 13(12–14) years. There was no difference in VPT, monofilament sensitivity and CCM measures between adolescents with obesity and controls. However, adolescents with AN had a significantly higher weight (P = 0.022), fat% (P = 0.029) and fat-muscle ratio (P = 0.012) with a lower CNFD (P = 0.045) compared to those with obesity without AN.

**Conclusion:**

Adolescents with obesity and acanthosis nigricans have a higher fat mass and small nerve fibre loss, indicative of a sub-clinical neuropathy.

## Introduction

The World Obesity Federation estimated that by 2025 there will be 206 million children and adolescents with obesity and this will increase to 254 million by 2030 [1]. The prevalence of childhood obesity varies from 7.9% in the UAE, 14.7% in Qatar, 15.8% in Saudi Arabia to 19.9% in Kuwait [2]. Childhood obesity is characterized by increased body fat mass with dyslipidemia, hypertension, insulin resistance (IR), impaired glucose tolerance (IGT) and type 2 diabetes mellitus (T2DM) [3, 4]. Acanthosis nigricans (AN) is characterized by thickened skin and brown pigmentation on the neck, axillae, knees and elbows and is indicative of underlying insulin resistance, metabolic syndrome and an increased risk of T2DM [5]. The severity of AN in children is associated with higher fasting insulin levels and Homeostatic Model Assessment for Insulin Resistance (HOMA-IR) score, indicative of insulin resistance [6].

Adults with impaired glucose tolerance (IGT) have evidence of neuropathy [7, 8] and children with obesity and IGT have abnormal nerve conduction studies (NCS) [4]. Children and adolescents with T2DM have abnormal pin prick, light touch and vibration perception threshold (VPT) [9, 10] and the incidence of neuropathy (VPT >20V) was two fold greater in children with T2DM (13.9/1000 patient years) compared to T1DM (7.8/1000 patient years) [11]. In the SEARCH study adolescents with T2DM had a higher age-adjusted prevalence of peripheral neuropathy (17.7% vs 8.5%) compared to T1DM [12]. Furthermore, the prevalence of DPN based on the Michigan Neuropathy Screening Instrument (MNSI) was 3-fold higher in youth with T2DM (22%) compared to T1DM (7%) and was associated with older age, longer duration of diabetes, smoking and lower HDL [13].

Corneal confocal microscopy has been used to identify early sub-clinical neuropathy and there is significant corneal and intra-epidermal nerve fiber loss in subjects with IGT [14] and recently diagnosed T2DM [15]. A lower corneal nerve fibre length also predicts the development of clinical DPN [16]. We have previously shown corneal nerve fiber loss in obese adults with and without diabetes and corneal nerve regeneration after bariatric surgery [17, 18]. The early identification of sub-clinical neuropathy is key to risk stratification and intervention to limit the development of overt and often irreversible clinical neuropathy. Given that AN is associated with an increased prevalence of insulin resistance and metabolic syndrome in children with obesity [19] we hypothesized there would be greater small nerve fibre damage in those with AN. Therefore, we assessed vibration perception threshold, monofilament sensitivity and CCM in adolescents with obesity with and without AN.

## Methods

### Participants and study design

This study evaluated 66 participants aged 12–17. Between April 2022 to March 2023, forty-six participants with obesity were recruited from the pediatric endocrinology clinic and 20 healthy controls were recruited from the general pediatric clinics in Sidra-Medicine, Qatar. Participants with a history of any other cause of neuropathy, malignancy, deficiency of B_12_ or folate, chronic renal failure, liver failure, connective tissue, or systemic disease (rheumatoid arthritis, systemic lupus erythematosus, dermatomyositis, systemic scleroderma, Raynaud phenomenon), previous corneal trauma or systemic disease affecting the cornea, and corneal surgery within 6 months of enrollment, were excluded. All participants provided written assent and parental informed consent and the research adhered to the tenets of Declaration of Helsinki and was approved by the Weill Cornell Medicine-Qatar (WCM-Q) (20–0006) and Sidra Medicine (1542992) Research Ethics Committees.

### Anthropometry

Weight (kg) was measured using the body composition analyzer (TANITA DC-430MAIII, Japan) and height (cm) using the stadiometer (SECA model, China) and both were recorded to the nearest 0.1 g or cm, respectively [20]. The cut-off points to classify weight status were established using the International Obesity Task Force (IOFT) [21] and WHO growth chart [22]. Body composition was only measured in participants with obesity using the TANITA scale following the manual input of height, gender, and age to derive the body fat percent (BF%), fat mass (kg), fat free mass (FFM) (kg), muscle mass (kg), fat-muscle ratio (FMR), total body water (TBW) (kg), TBW (%), and BMI (kg/m^2^). Height, weight, and BMI of the healthy control participants was measured as part of routine care and was collected from the electronic medical records. Participants with obesity were subdivided into those with and without acanthosis nigricans based on established criteria [23].

### Cardiometabolic panel assessments

Systolic (SBP) and diastolic (DBP) blood pressure, glycated hemoglobin (HbA1c), total cholesterol (TC), LDL cholesterol (LDL-C), HDL cholesterol (HDL-C), triglycerides (TG), and vitamin D (VitD) were assessed as part of routine care for all participants who attended the obesity clinic and data were collected from their electronic medical records.

### Neuropathy assessment

#### Vibration perception threshold (VPT)

Participants were asked to remove their shoes and socks and the stimulator was applied on the pulp of the big toe on both feet and the stimulus strength increased slowly from zero until the vibration was first perceived by indicating “yes”. Vibration sensation was recorded as an average for both feet in volts [24] and a VPT of **≥** 15V was considered to be impaired vibration perception [25].

#### Monofilament

A 10 g monofilament (Semmes-Weinstein monofilament, USA) was applied with a sufficient force to cause the filament to bend at a total of 9 sites per foot, on both feet and loss of protective sensation was recorded as “no feeling in **≥** 8 sites” [26].

#### Corneal confocal microscopy

Corneal confocal microscopy was undertaken using the Heidelberg Retina Tomograph III Rostock Cornea Module (Heidelberg Engineering, Heidelberg, Germany). Both eyes were anaesthetized with 2 drops of Bausch & Lomb Minims ^®^ (Oxybuprocaine hydrochloride 0.4% w/v). A drop of hypotears gel (Carbomer 0.2% eye gel) was placed on the tip of the objective lens and a sterile disposable TomoCap was placed over the lens, allowing optical coupling of the objective lens to the cornea. Six images were selected from the sub basal nerve plexus (SBNP) in the central cornea and corneal nerve fiber density (CNFD) (fibers/mm^2^) corneal nerve branch density (CNBD) (branches/mm^2^), and corneal nerve fiber length (CNFL) (mm/mm^2^) were quantified manually using CCMetrics. Six images centered on the inferior whorl and immediately adjacent area were selected and inferior whorl length (IWL) (mm/mm^2^) was quantified manually using the manual CNFL mode in CCMetrics. The investigator (HG) was blind to the study group when analyzing the CCM images.

### Statistical analysis

All statistical analyses were performed using IBM SPSS Statistics software Version 27 and P<0.05 was considered statistically significant. Normality of the data was assessed using the Shapiro-Wilk test and Q-Q plots. Data were expressed as mean ± standard deviation or median(range) based on their distribution. Comparison between healthy controls and adolescents with obesity or between adolescents with obesity with and without AN was performed using an independent t-test or Mann-Whitney U test, as appropriate. Multiple logistic lineaer regression was performed and the model included age to account for clinically and statistically significant risk factors for AN. Graph prism version 9 was used to build dot plots.

## Results

**Fig 1:**
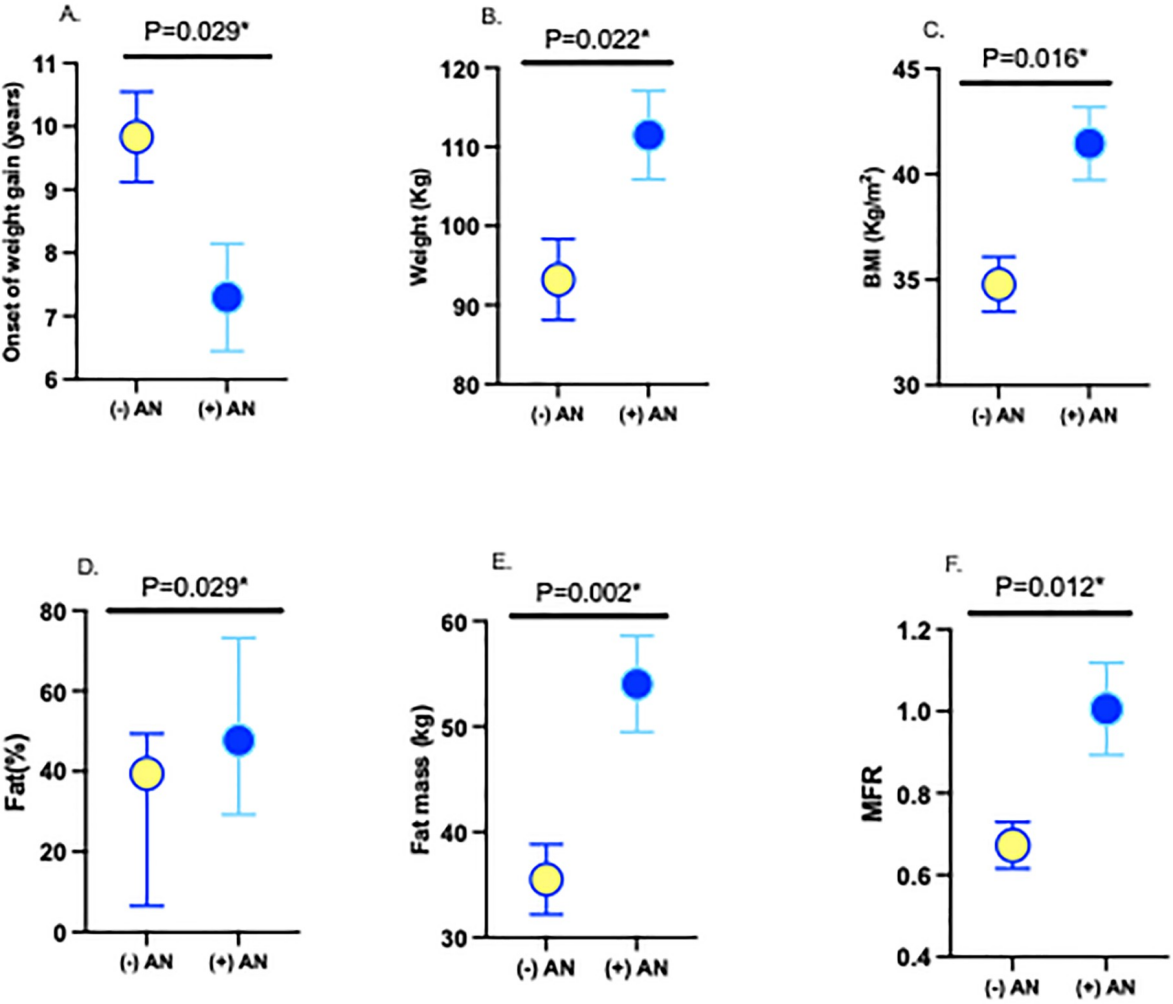
Body composition analysis in adolescents with obesity with and without AN, (A) onset of weight gain, (B) weight, (C) BMI, (D) Fat%, (E) Fat mass, (F) FMR. HC: healthy control; AN: acanthosis nigricans BMI: body mass index; FMR: fat-to-muscle ratio.

Forty-six participants with obesity (26 boys and 20 girls) aged 15(14–17) years were compared to 20 healthy controls. Obese adolescents were grossly overweight compared to healthy controls (weight 105.52±29.06 vs. 47.86±18.63, P<0.001) (Table 1). They were further sub-grouped into those with (n = 31) and without (n = 15) AN. There was no difference in systolic BP (P = 0.628), diastolic BP (P = 0.865), HbA1c (P = 0.823), total cholesterol (P = 0.636), LDL-C (P = 0.328), HDL-C (P = 0.711), triglycerides (P = 0.690) and vitamin D (P = 0.423) between adolescents with and without AN (Table 2). The weight of adolescents with AN was significantly higher than in those without AN (111.47±31.17 vs. 93.22±19.82, P = 0.022) and adolescents with AN gained weight at a younger age compared to those without AN (7.29±4.42 vs. 9.83±2.48, P = 0.029) (Fig 1A). BMI (41.45±9.62 vs. 34.79±5.06, P = 0.016), fat% (47.7(39.6–50.6) vs. 39.4(36.1–43.9), P = 0.029), fat mass (54.04±25.34 vs. 35.51±12.86, P = 0.002) and FMR (1.0±0.62 vs. 0.67±0.22, P = 0.012) (Fig 1A–1F) were significantly higher, with no significant difference in muscle mass (P = 0.809), FFM (P = 0.954), TBW (P = 0.755), TBW% (P = 0.098) between adolescents with and without AN (Table 2).

**Table 1:** Basic demographics and CCM parameters in adolescents with obesity compared to healthy controls.

Characteristics	Healthy Controls (n = 20)	Adolescents with Obesity (n = 46)	P-Value
Age (years)	13(12–14)	15(14–17)	0.014*
Weight (kg)	47.86±18.63	105.52±29.06	<0.001*
BMI (kg/m^2^)	22.26±5.47	39.27±8.92	<0.001*
CNFD (fiber/mm^2^)	27.81±7.11	30.09±7.36	0.247
CNBD (branch/mm^2^)	53.78±18.03	53.50±26.61	0.966
CNFL (mm/mm^2^)	20.53±4.48	21.92±5.82	0.347
IWL (mm/mm^2^)	25.21±4.56	23.69±5.85	0.333

HC: healthy control; body mass index; CNFD: corneal nerve fiber density; CNBD: corneal nerve branch density; CNFL: corneal nerve fiber length; C = IWL: inferior whorl length.

**Table 2:** Clinical, metabolic, body composition and neuropathy measures in adolescents with obesity with and without AN and significant differences between them.

Characteristics	(-) AN (n = 15)	(+) AN (n = 31)	P-Value	Adjusted P-Value
Age (years)	15(12.7–17.0)	15(14.0–16.5)	0.897	N/A
Age onset of weight gain (years)	9.83±2.48	7.29±4.42	0.029*	0.016*
Weight (kg)	93.22±19.82	111.47±31.17	0.022*	0.036*
SBP (mmHg)	114.54±9.26	112.77±11.57	0.628	0.604
DBP (mmHg)	73.16±6.05	72.73±7.87	0.865	0.863
HbA1c (%)	5.5(5.1–5.6)	5.4(5.3–5.6)	0.823	N/A
TC (mmol/L)	3.98±0.75	4.10±0.69	0.636	0.628
LDL-C (mmol/L)	2.54±0.77	2.76±0.62	0.328	0.311
HDL-C (mmol/L)	1.2(1–1.7)	1.1(1–1.4)	0.711	N/A
TG (mmol/L)	1.4(0.9–2.0)	1.0(0.8–1.4)	0.690	N/A
25 OHD (ng/mL)	41.69±23.40	35.55±20.61	0.423	0.399
Fat (%)	39.4(36.1–43.9)	47.7(39.6–50.6)	0.029*	N/A
Fat mass (kg)	35.51±12.86	54.04±25.34	0.002*	0.007*
Muscle mass (kg)	55.85±15.83	54.71±14.39	0.809	0.613
FFM (kg)	56.95±14.96	57.23±15.21	0.954	0.880
TBW (kg)	42.21±11.43	43.31±10.46	0.755	0.949
TBW (%)	44.4(41.9–47.2)	40.1(37.2–45.1)	0.098	N/A
BMI (kg/m^2^)	34.79±5.06	41.45±9.62	0.016*	0.011*
FMR	0.67±0.22	1.00±0.62	0.012*	0.033*
VPT (V)	2.85±0.98	3.00±1.04	0.626	0.607
CNFD (fiber/mm^2^)	33.19±7.13	28.58±7.09	0.045*	0.032*
CNBD (branch/mm^2^)	56.66±30.29	51.97±25.04	0.581	0.570
CNFL (mm/mm^2^)	23.29±5.76	21.25±5.82	0.270	0.251
IWL (mm/mm^2^)	24.21±6.94	23.49±5.51	0.749	0.744

*Significance at P<0.05

HC: healthy control; AN: acanthosis nigricans; SBP: systolic blood pressure; DBP: diastolic blood pressure; HbA1c: glycated hemoglobin; TC: total cholesterol; LDL-C: low-density-lipoprotein cholesterol; HDL-C: high-density-lipoprotein cholesterol; TG: triglycerides; 25 OHD: 25-hydroxy vitamin D; FFM: fat free mass; TBW: total body water; BMI: body mass index; FMR: fat-muscle ratio; VPT: vibration perception threshold; CNFD: corneal nerve fiber density; CNBD: corneal nerve branch density; CNFL: corneal nerve fiber length; IWL: inferior whorl length.

Adjusted model was for age and included the normally distributed variables.

### Peripheral neuropathy assessments

**Fig 2:**
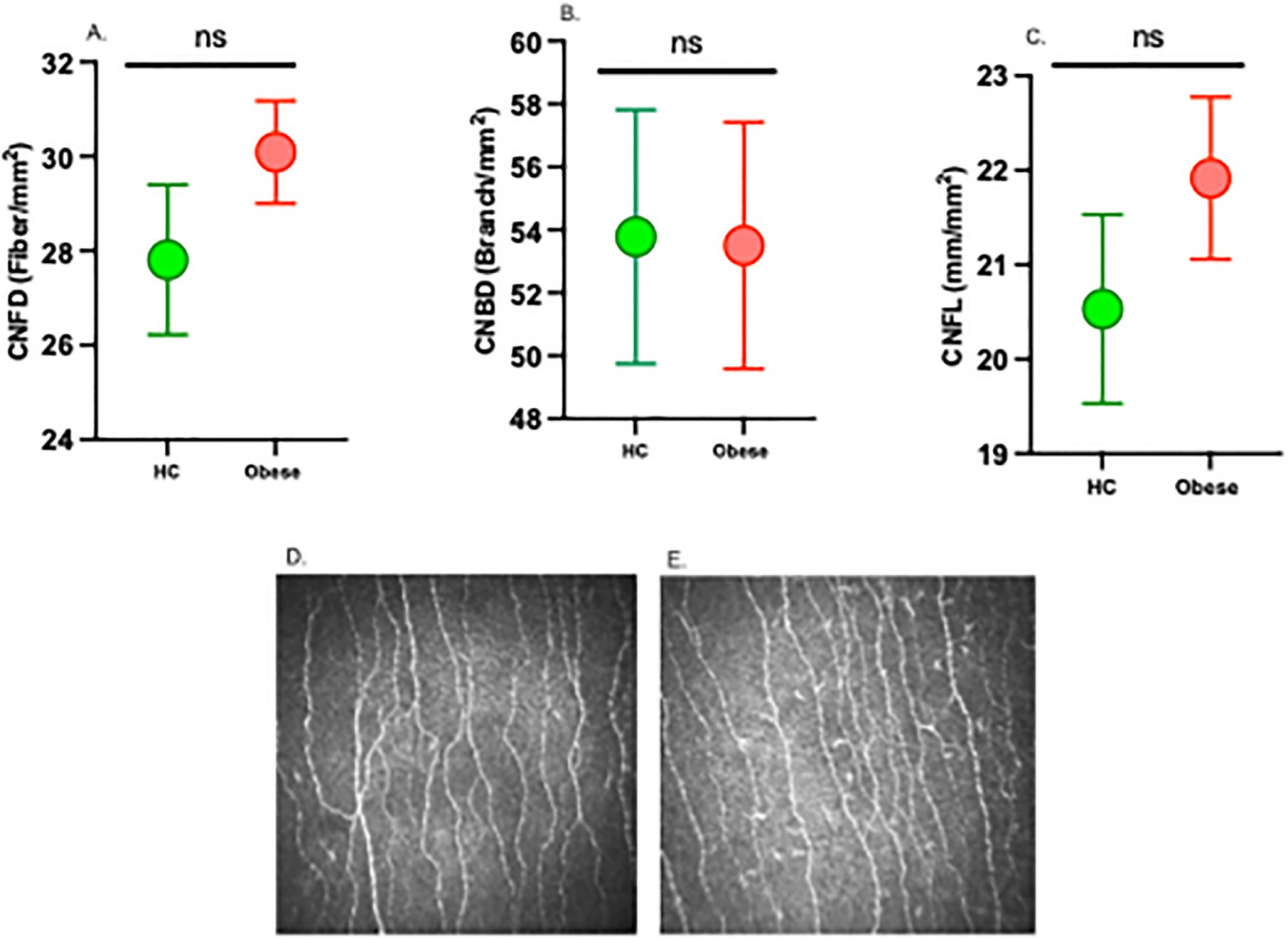
Corneal confocal microscopy measures (CNFD (A), CNBD (B) and CNFL (C)) and CCM image from a healthy control (D) and adolescent with obesity (E). CNFD: corneal nerve fiber density; CNBD: corneal nerve branch density; CNFL: corneal nerve fiber length.

There was no significant difference in VPT, sensitivity to the monofilament, CNFD, CNBD, CNFL and IWL in adolescents with obesity compared to HC (Fig 2A–2E).

**Fig 3:**
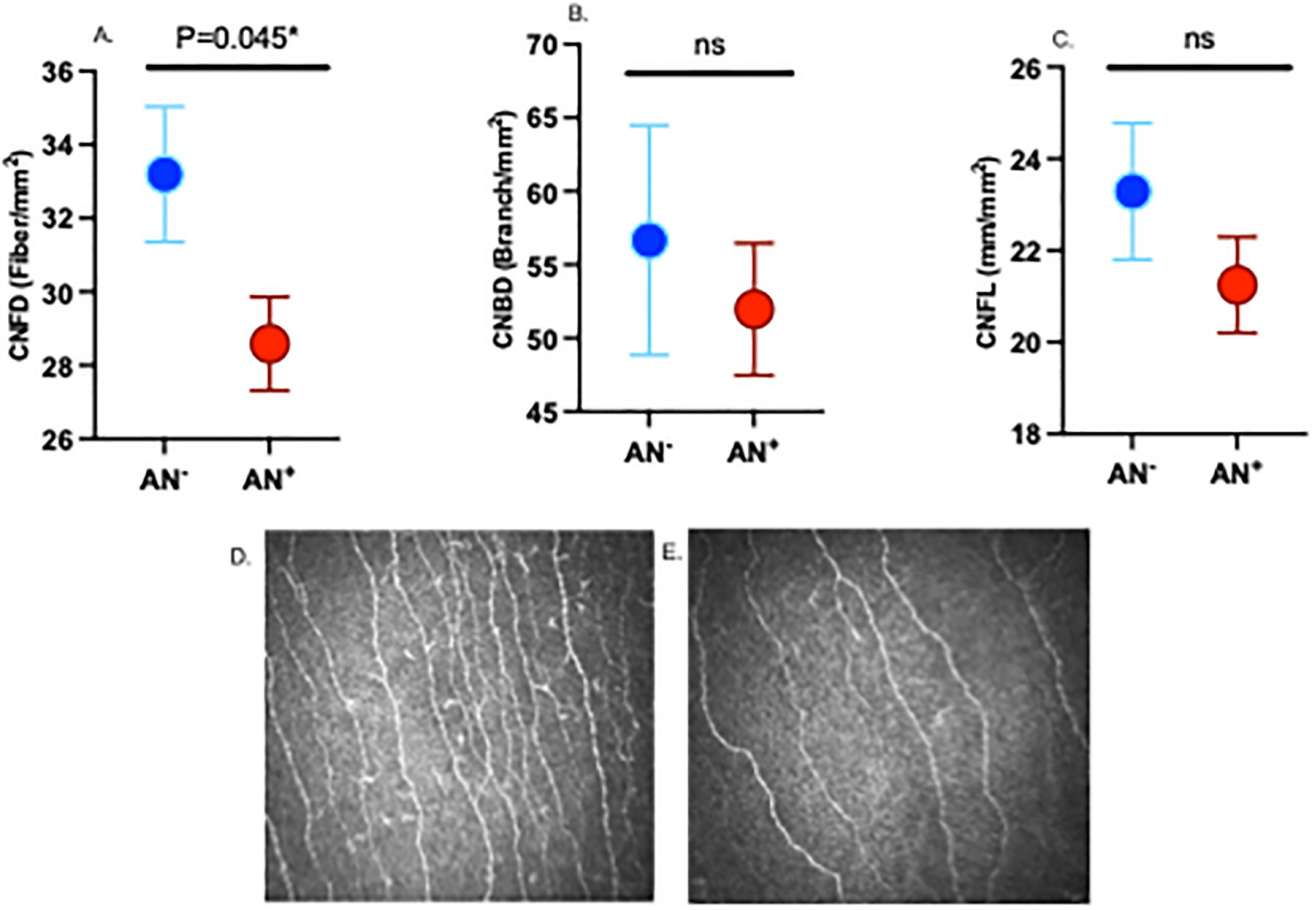
Corneal confocal microscopy measures (CNFD (A), CNBD (B) and CNFL (C)) and CCM image from an adolescent with obesity without AN (D) and a child with obesity and AN (E). AN: acanthosis nigricans; CNFD: corneal nerve fiber density; CNBD: corneal nerve branch density; CNFL: corneal nerve fiber length.

There was no significant difference in vibration perception threshold (VPT) or monofilament insensitivity between adolescents with obesity with and without AN. CNFD was significantly lower, whilst CNBD, CNFL and IWL were comparable in adolescents with obesity with and without AN (Fig 3A–3E).

## Discussion

The present study shows that adolescents with obesity and acanthosis nigricans have evidence of corneal nerve loss, indicative of a sub-clinical small fibre neuropathy. These adolescents with AN also have greater weight, percentage body fat and fat-muscle ratio compared to those without AN, consistent with previous studies in adults and children with AN [5, 27]. A higher fat-muscle ratio is associated with fatty liver disease, metabolic syndrome and insulin resistance [28] and high percentage body fat is associated with insulin resistance, higher triglycerides, visceral fat mass [29] and glucose dysregulation with a higher risk of developing T2DM [30]. Indeed, the American Diabetes Association (ADA) has recognized AN as a formal risk factor for developing T2DM [31].

Adults with insulin resistance have evidence of blunted corneal nerve regeneration following an improvement in glycemic control [32]. Furthermore, an abnormal lipid profile is associated with neuropathy in subjects with IGT [33] and a higher BMI, total cholesterol and VLDL cholesterol are associated with neuropathy in patients with diabetes [34]. High-fat-diet fed (HFD) rats have lower levels of synapsin I protein, important for neurotransmission and neuronal plasticity [35] and inflammation mediated by long-chain fatty acids (LCFAs) [36] which induces Schwann cell endoplasmic reticulum (ER) dysfunction, mitochondrial depolarization and generation of reactive oxygen species [37]. There is an increasing body of evidence linking lipid abnormalities to neuropathy [38] and we have previously shown that adults with obesity and abnormal lipoproteins and HDL functionality have evidence of corneal nerve loss [39, 40]. Indeed, bariatric surgery was associated with an improvement in lipoprotein oxidation [41] and glycation [42], HDL functionality [43], inflammation and insulin resistance [44] with corneal nerve regeneration [17, 18]. We have also shown that subjects with impaired glucose tolerance [14], especially those with greater insulin resistance who develop type 2 diabetes [45] have greater corneal nerve loss. In the current study, adolescents with obesity and AN did not differ from those with obesity without AN in relation to their blood pressure, lipid profile and HbA1c, but they had increased fat mass and there is an increasing body of evidence showing that increased visceral adiposity and alterations in adipokines are associated with diabetic neuropathy [46–50].

We acknowledge this study is cross-sectional in design and therefore cause effect cannot be established and it had a small sample size. Furthermore, we have not undertaken an objective assessment of insulin resistance e.g. HOMA-IR or measurement of adipokines. Nevertheless, this is the first study to show that adolescents with obesity perse have normal corneal nerve morphology, but those with acanthosis nigricans have evidence of early small nerve fibre degeneration. These observations warrant larger longitudinal studies to assess if AN associated corneal nerve loss can predict the development of neuropathy in adolescents with obesity.

## Supporting information

S1 Dataset The file includes clinical characteristics, body composition analysis and CCM data. (XLSX)
